# PPARs and the Development of Type 1 Diabetes

**DOI:** 10.1155/2020/6198628

**Published:** 2020-01-09

**Authors:** Laurits J. Holm, Mia Øgaard Mønsted, Martin Haupt-Jorgensen, Karsten Buschard

**Affiliations:** The Bartholin Institute, Department of Pathology, Copenhagen University Hospital, Denmark

## Abstract

Peroxisome proliferator-activated receptors (PPARs) are a family of transcription factors with a key role in glucose and lipid metabolism. PPARs are expressed in many cell types including pancreatic beta cells and immune cells, where they regulate insulin secretion and T cell differentiation, respectively. Moreover, various PPAR agonists prevent diabetes in the non-obese diabetic (NOD) mouse model of type 1 diabetes. PPARs are thus of interest in type 1 diabetes (T1D) as they represent a novel approach targeting both the pancreas and the immune system. In this review, we examine the role of PPARs in immune responses and beta cell biology and their potential as targets for treatment of T1D.

## 1. Introduction

T1D is an autoimmune disease caused by the pancreatic beta cells being dysfunctional or killed by autoreactive T cells resulting in reduced insulin production and hyperglycemia [1, 2]. The incidence of T1D is increasing, and estimates from the International Diabetes Federation suggests that the number of patients (age < 20 years) has doubled from 2015 to 2017 [3, 4]. However, the incidence varies geographically with high rates in Finland (>60 cases/100.000/year) and Sardinia (~40 cases/100.000/year), while China has less than one case/100.000/year [5]. The strongest genetic susceptibility is the HLA haplotypes DR3-DQ2 and DR4-DQ8 with 90% of diagnosed children having one or both haplotypes in Scandinavia [6, 7]. Over 50 genetic loci contribute to the genetic disease predisposition, although the molecular mechanisms often remain unknown [8]. Less than 10% of genetically susceptible individuals develop T1D, demonstrating that environmental factors such as diet and microorganisms play a pivotal role in T1D pathology [9, 10]. It was previously believed that patients had an almost complete loss of beta cells at onset of disease. However, several recent studies have shown that new-onset T1D patients retain up to 40% of insulin-positive islets [11–13]. Furthermore, islets isolated from T1D patients can regain their ability to secrete insulin when cultured in a nondiabetogenic environment *in vitro* [14]. Thus, beta cell dysfunction is likely to play an important role in T1D pathology. Current therapeutic approaches have, with limited clinical efficacy, focused on suppressing the ongoing immune attack or stimulating beta cell regeneration [15, 16]. Therefore, strategies that both dampen the immune response and promote beta cell function are in high need. The PPAR family is an ideal target for such a strategy, as PPARs have both anti-inflammatory properties, regulate beta cell biology, and modulate the pancreatic lipidome.

## 2. PPARs

**Figure 1:**
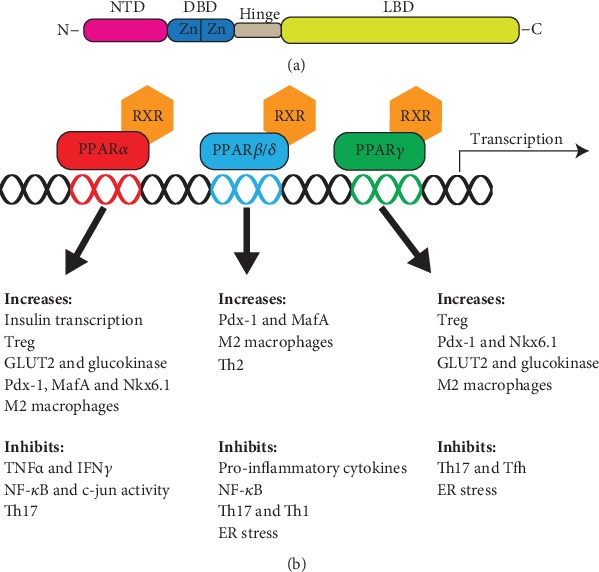
Structure and function of PPARs. (a) The peroxisome proliferator-activated receptor (PPAR) isoforms have a large degree of structural overlap, consisting of an N-terminal ligand-independent transactivation domain (NTD). The DNA-binding domain (DBD) contains two zinc finger (Zn) domains, which bind to peroxisome proliferator response element (PPRE) sequences. The DBD is connected through a hinge domain to the C terminal ligand-binding domain (LBD). (b) Illustration of the biological role of PPARs. PPARs heterodimerize with members of the retinoid X receptor (RXR) family. The isoforms are involved in a variety of pathways; shown are pathways with relation to type 1 diabetes. c-jun: transcription factor c*-*Jun; GLUT2: glucose transporter 2; MafA: MAF bZIP transcription factor A; NF*κ*B: nuclear Factor-kB; Nkx6.1: NK6 homeobox 1; Pdx-1: pancreatic and duodenal homeobox 1; Tfh: follicular helper T cells; Th1: T helper 1 cells; Th17: T helper 17 cells; Th2: T helper 2 cells; TNF*α*: tumor necrosis factor alpha; Treg: regulatory T cells.

PPARs were identified in the 1990s as mediators of peroxisome proliferation [17]. They belong to the nuclear receptor class II superfamily of transcription factors and regulate a range of biological processes by modulating gene expression. In mammals, three isoforms have been identified: PPAR*α* (NR1C1), PPAR*β*/*δ* (NR1C2), and PPAR*γ* (NR1C3), which predominately control genes involved in lipid metabolism including transport, storage, lipogenesis, and fatty acid oxidation (FAO) [17]. PPARs are important targets for metabolic disorders and multiple drugs targeting PPAR*α* (fibrates, e.g., fenofibrate, bezafibrate, and clofibrate) and PPAR*γ* (thiazolidinediones, e.g., troglitazone, rosiglitazone, pioglitazone, and ciglitazone) which have been used to treat hyperlipidemia and type 2 diabetes. PPARs are dynamic as they shuttle between the nucleus and cytoplasm, though they are mainly and constitutively present in the nucleus [18, 19]. The nuclear-cytoplasmic shuttling of PPARs is regulated by binding of PPAR ligands to the C-terminal domain (Figure 1) [19]. Binding of ligands induces a conformational change leading to heterodimerization with members of the retinoid X receptor (RXR) family [20, 21]. This complex binds to specific DNA sequences, termed peroxisome proliferator response elements (PPRE) through the highly conserved zinc finger DNA-binding domain in the N terminus [22]. Binding of ligands also results in dissociation of corepressors and recruitment of coactivator proteins, resulting in enhancement of target gene transcription [23]. In the absence of ligands, PPARs instead recruit corepressors that repress transcription of target genes [24]. PPARs are involved in a mechanism termed “transrepression,” which is a ligand-dependent but PPRE-independent mechanism of gene repressions through interactions with other proteins such as NF*κ*B, AP1, and STAT [25–27]. This generates and stabilizes corepressing complexes, which typically bind to and repress proinflammatory genes [21].

The PPAR isoforms have a great degree of structural and functional overlap but their expression patterns differ. PPAR*α* is highly expressed in metabolically active tissues including liver, kidney, and adipose tissue. PPAR*α* is activated during fasting and is involved in controlling ketogenesis, lipoproteins, gluconeogenesis, amino acid catabolism, FAO, and inflammatory responses [28]. PPAR*β*/*δ* is nearly ubiquitously expressed and involved in FAO and activation has an anti-inflammatory effect with reduced secretion of proinflammatory cytokines [29]. PPAR*γ* is expressed in various tissues including adipose, intestine, liver, and kidney [30, 31]. It is involved in regulating fat cell differentiation, lipid storage, and differentiation of monocytes into macrophages [32, 33]. PPARs have, due to their immune regulatory functions, been linked to several autoimmune diseases, i.e., multiple sclerosis [34], lupus erythematosus [35], autoimmune thyroiditis [36], Graves ophthalmopathy [37], rheumatoid arthritis [38], psoriasis [39], and Guillain–Barré [40]. Similarly, PPARs have also been suggested as targets to treat chronic inflammatory diseases [20, 41]. An interesting feature is that women seem to be more susceptible than men to develop autoimmune diseases [42]. This might be connected to PPAR expression as mouse studies have found that male mice have higher expression of PPAR*α* in T cells compared to female mice, and that expression was androgen sensitive [43].

Polymorphisms in PPAR*β*/*δ* and PPAR*γ* promoter regions contribute to the genetic predisposition to T1D and affect the severity of islet autoimmunity [44]. Additionally, PPAR*γ* is associated with the development of insulin resistance and type 2 diabetes [45].

## 3. PPARs and the Immune System

The pathogenesis of T1D includes interactions between beta cells and components of both the innate and adaptive immune system [46]. Many different immune cells have been implicated including B cells and macrophages [47, 48]. However, focus has primarily been on T cells where evidence suggests that T1D develops due to a defect in regulatory T cell (Treg) function [2, 46]. Studies of postmortem pancreas samples from T1D patients revealed that CD8^+^ T cells are the most predominant population in the islet infiltrate followed by (in declining order) macrophages, CD4^+^ T cells, B cells, and plasma cells [49]. Why tolerance is lost in some individuals remains unknown.

The metabolic pathway for ATP production has an important role in regulating immune cell function. Differentiation of activated CD4^+^ T cells thus depends on the metabolic pathway; Th1, Th2, and Th17 cells use glycolysis while Tregs have a high level of lipid oxidation [50, 51]. In this way, T cell differentiation can be manipulated as inhibition of glycolysis blocks Th17 and promotes Treg differentiation [51]. The inflammatory M1 phenotype of macrophages uses glycolysis while the anti-inflammatory M2 phenotype utilizes lipid oxidation [52]. Hence, modulation of FAO through PPARs can induce immunological changes. PPARs are expressed in various types of immune cells including macrophages, dendritic cells, B cells, and T cells, and all three isoforms have anti-inflammatory activities [53]. Activation of all PPARs potentiates the polarization of mouse macrophages to the anti-inflammatory M2 phenotype, while M2 is diminished in PPAR*γ* and PPAR*β*/*δ* knockouts [20, 32, 54, 55]. Deletion of PPAR*γ* in macrophages blocks FAO and renders the macrophages incapable of making a full conversion to the M2 phenotype. Only PPAR*γ* seems to have the same role in human macrophages [20]. This anti-inflammatory effect appears to depend on the repression of NF*κ*B and AP-1 [20, 54, 56, 57].

The role of PPARs in T cell regulation is more complex with differences between the isoforms. Tregs from PPAR*α* knockout mice have impaired suppressive activities towards both CD4^+^ and CD8^+^ T cells [58]. This was associated with reduced migratory abilities and diminished expression of several chemokine receptors. In support of this, PPAR*α* knockout mice have prolonged inflammatory response to inflammatory agents such as arachidonic acid [59]. The PPAR*α* agonist fenofibrate has been demonstrated to promote FOXP3^+^ regulatory T cells in mice [60, 61]. PPAR*α* is involved in regulating effector T cells with high expression of PPAR*α* leading to increased production of Th2 cytokines and knockout mice having increased differentiation towards a Th1 phenotype [43]. Also, fenofibrate treatment prevented the differentiation of Th17 cells in mice [62]. In addition, PPAR*α* agonist WY14643 diminishes human T cell proliferation and induce T cell depletion by trapping the cells in the G2/S phase [63]. In hyperlipidemia patients, treatment with fenofibrate decreases TNF*α* and IFN-*γ* levels [64]. These findings were validated in PPAR*α* knockout mice as they had increased levels of TNF*α* and IFN-*γ* [43].

PPAR*β*/*δ* activation inhibits Th1 and Th17 while enhancing Th2 [65–67]. Deletion of PPAR*β*/*δ* gives the opposite result. This is likely a consequence of PPAR*β*/*δ* increasing FAO [68], thereby blocking the proliferative burst following antigen recognition in T cells as a consequence of a shift from oxidative metabolism to glycolysis [20, 69].

PPAR*γ* seems to have a role in regulating the balance between regulatory and effector T cells. Reduced PPAR*γ* activity increases the amount of effector T cells as evidenced by increased antigen-specific proliferation and overproduction of IFN-*γ* in response to IL-12 in PPAR*γ* knockout mice [70]. There is also evidence indicating that PPAR*γ* inhibits expression of ROR*γ*t and thereby differentiation of Th17 cells in both mice and humans [71]. PPAR*γ* appears to be involved in the formation of follicular helper T cells (Tfh) as mice with a knockout in CD4 cells had increased Tfh cell activation and increased formation of germinal centers [72]. PPAR*γ* agonist troglitazone and rosiglitazone have additional in a mouse model of colitis been shown to shift the immune response from Th1 towards Th2, with a corresponding decrease in Th1-associated transcription factors, cytokine and chemokine, and an increase in Th2-associated factors [73, 74]. On the other hand, PPAR*γ* deficiency leads to a decreased number of CD4^+^FOXP3^+^ regulatory T cells [75]. This is exemplified by the identification of a specific Treg population with a high expression of PPAR*γ* in visceral adipose tissue [76]. PPAR*γ* is the major orchestrator of these Tregs, and Treg-specific deletion of PPAR*γ* prevented the formation of this cell type. Furthermore, the loss of PPAR*γ* in Tregs leads to increased effector T cell responses while PPAR*γ* activation increases the amount of FOXP3^+^ regulatory T cells [70, 75, 77]. Another study has though described how rosiglitazone had no effect on Tregs in a mouse model of allergic asthma [78], thereby suggesting the effect of PPAR*γ* on Tregs might be tissue-specific.

## 4. PPARs and Pancreatic Islets

Beta cells are highly specialized cells each making millions of insulin molecules per day [79]. This puts tremendous pressure on the cells, as insulin is prone to misfolding with approximately 20% of all insulin molecules failing to reach its mature conformation [80]. Misfolded insulin can lead to ER stress, which again can lead to the formation of neoantigens and activate the immune system resulting in further beta cell death and loss of insulin production [81]. As described above, beta cell dysfunction rather than beta cell death has recently been emphasised as a major contributor to T1D. Thus, the possibility of restoring beta cell function has become an alluring research area. In this regard, the PPAR isoforms are possible targets as they are expressed in pancreatic islets [82–84] and appear to have important roles as regulators of beta cell biology.

PPAR*α* is expressed in pancreatic islets and beta cell lines with expression depending on glucose level [85]. High glucose represses PPAR*α* in isolated rat islets and INS-1E cells [86, 87]. The glucose-dependent upregulation of insulin expression might rely on PPAR*α* as glucose did not increase insulin expression in islets from PPAR*α* knockout mice [88]. PPAR*α* knockout mice have reduced mRNA levels of insulin, Nkx6.1 (a transcription factor essential for maintaining functionally mature beta cells [89]), MafA (regulator of insulin secretion [90]), GLUT2, and glucokinase [91]. PPAR*α* has likewise been found to upregulate Pdx-1 (transcription factor with a critical role in pancreas and beta cell development [92]) in INS-1 cells and isolated rat islets [93, 94]. On a whole-body level, PPAR*α* knockout mice are normoglycemic in a fed state but hyperglycemic when fasted [85]. This was associated with a 55% higher plasma insulin level. The mice had improved glucose tolerance and increased insulin secretion from isolated islets.

PPAR*β*/*δ* is the most abundant PPAR isoform in beta cells [83, 95]; however, not much is known about its role in beta cell biology. PPAR*β*/*δ* appears to have an important role in pancreas development as pancreatic PPAR*β*/*δ* knockout mice had an increased number of pancreatic islets and a 2-fold increase in beta cell mass [96]. This was associated with increased plasma insulin levels, hypoglycemia, and improved glucose tolerance, while isolated islets had an increased second-phase insulin secretion. This suggests that PPAR*β*/*δ* is a negative regulator of insulin secretion in the mature pancreas, which is in contrast to a study demonstrating that PPAR*β*/*δ* promotes beta cell differentiation from stem cells by upregulating Pdx-1 [97]. GW501516, a PPAR*β*/*δ* agonist, was shown to attenuate dysfunction of palmitate-induced insulin secretion by promoting MafA [98]. Furthermore, this agonist promoted FAO and protected against palmitate-induced ER stress in a beta cell line [99]. PPAR*β*/*δ* was also demonstrated to reduce ER stress in rodent models [100, 101]. Additionally, GW501516 improved beta cell mitochondrial function in Desnutrin knockout mice and reduced lipolysis, which resulted in improved glucose tolerance and glucose-stimulated insulin secretion (GSIS) [95].

The role of PPAR*γ* in insulin secretion is not fully understood. Some studies have demonstrated that PPAR*γ* activation or overexpression suppresses insulin secretion and proinsulin biosynthesis [102–106]. For example, it was shown that overexpressing PPAR*γ* in INS-1E cells leads to impairment of GSIS [105]. However, other studies have demonstrated that PPAR*γ* activation or overexpression potentiates GSIS in beta cells and isolated islet [107–110]. What we do know is that PPAR*γ* is involved in controlling several key beta cell genes. Activation of PPAR*γ* by troglitazone (a PPAR*γ* agonist) leads to upregulation of Pdx-1, Nkx6.1, glucokinase, and GLUT2 [111, 112]. In addition, PPAR*γ* pancreatic knockout mice had reduced Pdx-1 protein levels in islets [113]. This is supported by findings of PPRE sequences in the promoter region of GLUT2 [114], glucokinase [115], and Pdx-1 [111, 113]. Furthermore, troglitazone was demonstrated to increase the half-life of Pdx-1 and MafA by inhibiting ubiquitination, which otherwise targets them for degradation by the proteasome [116]. The role of PPAR*γ* in pancreas development is not completely understood as PPAR*γ* pancreatic knockout mice are hyperglycemic despite having a normal pancreas morphology [113]. *In vivo* studies found that long-term rosiglitazone (a PPAR*γ* agonist) or troglitazone treatment maintains beta cell proliferation and prevents the age-related loss of pancreatic mass in rats and mice [117–119]. Troglitazone can also prevent age-related pancreatic abnormalities and increases in fasting insulin levels [120, 121].

Other studies have shown that PPAR*γ* agonists improve beta cell function and prevent mitochondrial alterations and diabetes in obese mice and rats [117, 118, 122]. In addition, activation of PPAR*γ* protects against cytokine-induced apoptosis [123], lipotoxicity [124], and human islet amyloid polypeptide toxicity [125, 126]. A molecular explanation for these findings might be that activation of PPAR*γ* is associated with a reduced amount of reactive oxygen species by inhibiting iNOS through NF*κ*B [123]. PPAR*γ* activation reduces islet ER stress in db/db mice and a diabetic ER stress mouse model [112, 127].

## 5. PPARs Regulate Sphingolipid Metabolism

We have recently described how the onset of T1D is associated with an abnormal sphingolipid metabolism in pancreatic islets. This was illustrated by newly diagnosed T1D patients having a reduced amount of the sphingolipid sulfatide and altered expression of several enzymes involved in sphingolipid metabolism in islets [44]. Sphingolipid metabolism is also altered before the onset of diabetes. Peripheral blood mononuclear cells from children progressing to T1D have altered levels of several sphingolipid species and altered expression of genes involved in sphingolipid metabolism [128]. PPAR*α* is known to control the expression of cerebroside sulfotransferase (CST), which catalyses the last step in sulfatide biosynthesis. PPAR*α* knockout mice had decreased CST expression associated with decreased serum sulfatide [129]. PPAR*α* activation by fenofibrate leads to increased sulfatide concentration in the pancreas and multiple other organs [44, 130, 131]. This was associated with an increased CST expression in the corresponding tissue [130, 131]. Similarly, fatty acids have been shown to activate PPAR*α* and increase sulfatide levels through SPTLC2 (subunit of serine palmitoyltransferase), which regulates the first step in sphingolipid synthesis [132]. Treatment with PPAR*α* agonist WY14643 or bezafibrate leads to increased expression of SPTLC2 in various cell types [133–135]. SPTLC2 and CST both have PPAR*α* binding sequences in their promoter region [132]. PPAR*α* is similarly involved in regulating the composition of sulfatide species with C16 (insulin folding and secretion) and C24 (immune regulation) having different functions [136, 137]. In the pancreas, fenofibrate especially increased the amount of C24 sulfatide thereby creating an anti-inflammatory sulfatide composition [138].

Another sphingolipid with a suspected role in T1D pathology is the proapoptotic ceramide of which C16 promotes apoptosis, mitochondrial dysfunction, and insulin resistance [139–142], while C24 has beneficial roles in regulating metabolic health [141, 143]. Recently, we demonstrated that fenofibrate altered ceramide composition in the pancreas of NOD mice increasing C24 and decreasing C16, hence creating a more beneficial ceramide composition [138]. WY14643 was otherwise found to increase ceramide levels in rat hearts [134], suggesting organ-specific regulation of ceramide synthesis. PPAR*β*/*δ* and PPAR*γ* are both known to regulate sphingolipid metabolism with PPAR*β*/*δ* agonist GW0742 and PPAR*γ* agonist troglitazone increasing *de novo* synthesis in rat hearts [144].

## 6. PPAR Activation Prevents Diabetes in NOD Mice

NOD mice share many autoantigens and biomarkers with human patients, and much has been learned from this model concerning the identification of genetic and environmental risk factors [145]. Experiments on NOD mice are primarily performed on females owing to a diabetes incidence of approximately 80%, compared to approximately 20% in males [146]. The higher incidence in females might be connected to the gender-specific changes in the expression of PPAR*α* and PPAR*γ*. Female NOD mice had increased expression of PPAR*α*, while PPAR*γ* was decreased in macrophages and CD4^+^ lymphocytes compared to male NOD mice [147]. Additionally, NOD mice have altered expression of PPAR*α* and PPAR*γ* in CD4^+^ or CD8^+^ lymphocytes and macrophages compared to non-obese diabetic-resistant (NOR) mice [148].

We and others have demonstrated that activation of PPAR*α* by fenofibrate or PPAR*γ* by troglitazone and rosiglitazone results in reduced autoimmune diabetes incidence [44, 149]. Fenofibrate treatment initiated after disease onset could even reverse diabetes in 46% of female NOD mice [44]. In addition, troglitazone prevents hyperglycemia and reduces insulitis in mice following streptozotocin injections [150]. PPARs are also regulated by various naturally occurring agonists, of which several have been examined for their effect on autoimmune diabetes in NOD mice (Table 1). This includes epigallocatechin [151, 152], curcumin [153, 154], cannabidiol [155, 156], omega 3 fatty acids [157], and capsaicin [158, 159], which induce PPAR activity and protect against autoimmune diabetes in NOD mice.

Taurine, which stimulates PPAR*α*, in the diet during gestation and lactation reduces diabetes development in offspring of NOD mice [160, 161]. On a similar note, a gluten-free diet, which leads to increased expression of PPAR*α* and PPAR*γ* [162], was found to reduce diabetes incidence in NOD mice [163], even after exclusive exposure of the diet in utero [164, 165].

## 7. Conclusions

Numerous studies have examined PPARs in relation to their role as regulators of lipid metabolism. However, the isoforms are also potent regulators of inflammation and beta cell biology (Figure 1). The effects of PPAR activation on T cell survival, activation, and differentiation are likely beneficial in a T1D setting but remain unstudied to a large extent. The same is true for studies of pancreas biology with most studies being conducted in relation to type 2 diabetes. Thus, we need further studies to determine the precise role of PPARs in T1D pathology. The beneficial effect on NOD mice by PPAR agonists is promising, and we believe that modulation of PPARs represents a novel treatment strategy targeting both the immune system and the pancreas.

## Figures and Tables

**Table 1:** Overview of treatments that promote PPAR expression and prevent autoimmune diabetes in NOD mice.

Drug	Delivery	Diabetes incidence	Reference
Fenofibrate	Diet from age 3 weeks	0%	[44]
Troglitazone	Oral gavage from age 3 weeks	22%	[149]
Rosiglitazone	Oral gavage from age 3 weeks	22%	[149]
Epigallocatechin	Water from age 5 weeks	25%	[151]
Curcumin	i.p. every other day	33 % (CYP-induced diabetes)	[153]
Cannabidiol	i.p. age 6-12 weeks	30%	[155]
Capsaicin	Oral gavage at age 9 or 10 weeks	20%	[158]
Taurine	Water to pregnant mothers	40% (after 24 weeks)	[160]
Omega-3	Diet from age 5 weeks	33%	[157]
Gluten-free diet	Diet from breeding	15%	[163]
